# Source apportionment to support air quality planning: Strengths and weaknesses of existing approaches

**DOI:** 10.1016/j.envint.2019.05.019

**Published:** 2019-09

**Authors:** P. Thunis, A. Clappier, L. Tarrason, C. Cuvelier, A. Monteiro, E. Pisoni, J. Wesseling, C.A. Belis, G. Pirovano, S. Janssen, C. Guerreiro, E. Peduzzi

**Affiliations:** a European Commission, Joint Research Centre, Ispra, Italy; b Université de Strasbourg, Laboratoire Image Ville Environnement, Strasbourg, France; c NILU - Norwegian Institute for Air Research, Kjeller, Norway; d Ex European Commission, Joint Research Centre, Ispra, Italy; e CESAM, Department of Environment and Planning, University of Aveiro, Aveiro, Portugal; f RIVM, National Institute for Public Health and the Environment, Bilthoven, the Netherlands; g RSE Spa, Milano, Italy; h VITO, Boeretang 200, 2400 Mol, Belgium

**Keywords:** Urban air pollution, Source allocation, Source apportionment, Increment, Contribution, Impact, Air quality planning, Brute force, Mitigation strategy

## Abstract

Information on the origin of pollution constitutes an essential step of air quality management as it helps identifying measures to control air pollution. In this work, we review the most widely used source-apportionment methods for air quality management. Using theoretical and real-case datasets we study the differences among these methods and explain why they result in very different conclusions to support air quality planning. These differences are a consequence of the intrinsic assumptions that underpin the different methodologies and determine/limit their range of applicability. We show that ignoring their underlying assumptions is a risk for efficient/successful air quality management as these methods are sometimes used beyond their scope and range of applicability. The simplest approach based on increments (incremental approach) is often not suitable to support air quality planning. Contributions obtained through mass-transfer methods (receptor models or tagging approaches built in air quality models) are appropriate to support planning but only for specific pollutants. Impacts obtained via “brute-force” methods are the best suited but it is important to assess carefully their application range to make sure they reproduce correctly the prevailing chemical regimes.

## Highlights

• Different source-apportionment approaches may lead to different conclusions to support air quality planning.
• The incremental approach is generally not suited to support air quality planning.
• Receptor models or tagging approaches are appropriate to support planning but only for specific pollutants.
• “Brute-force” methods are the best suited but their application range must be assessed.

## 1 Introduction

Air pollution is one of the main causes of damages to human health in Europe, with an estimate of about 390,000 premature deaths per year in the EU28, as the result of exposure to fine particulate matter (PM_2.5_) only (EEA, 2018). Twenty-six out of twenty-eight European Union countries yet fail to comply with at least one of the limit values set by the EU air quality directives in 2016, in particular for PM. Many of the exceedances to the EU limit values for PM occur in urban areas where most of the population lives. One of the main challenges to improve this situation is to understand the origin of the pollution to make sure that air quality plans are targeting the appropriate sources at the right scale to ensure effective results.

Air quality plans involve, among others, the following tasks: (1) identify and quantify the sources that contribute most to concentration levels; (2) inform on the efficiency of mitigation strategies; (3) identify possible measures to be applied to each of these sources and/or (4) evaluate scenarios for future emissions to assess the effectiveness of mitigation measures to control air quality levels. Source apportionment methodologies aim at understanding the origin of the pollution and are generally used for the two first inter-related tasks. This work focuses more specifically on the use of source apportionment to support the second task.

While EU wide air quality policies have been effective in reducing widespread levels of pollution, “hotspots” regions remain in the Po-valley and Eastern Europe for particulate matter, in most of southern Europe for ozone and in most cities in the whole of the EU for nitrogen dioxides (EEA, 2018). Given this “hotspot” situation, source apportionment becomes a key instrument to support local, regional and national authorities in designing effective air quality plans. This is why reliable information on the origin of pollution and quantification of the responsibility of different sources to pollution levels is requested by the European Air Quality Directive (2008/50/EC). However, the European Court of Auditors (ECA, 2018) recently raised the issue that air quality plans (AQP) were not designed as “effective tools” because they were short of targeted measures and plans could not be implemented quickly enough for the areas where the highest concentrations were measured. Part of the issue may arise from the fact that different source-apportionment approaches lead to results that generally differ among themselves, characterized by important under- or over-estimation of the role of specific sectors or spatial sources (Burr and Zhang, 2011b; Burr and Zhang, 2011a; Kranenburg et al., 2013; Clappier et al., 2017; Thunis, 2018). These under- or over-estimations can lead to incorrect conclusions about the responsibility of given sectors and about the efficiency of mitigation strategies.

Based on simplified theoretical examples, Clappier et al. (2017) and Grewe et al. (2010) clearly made the point that some methods were conceptually designed to address different questions and in particular that some methods were not always suited to inform on the impact of emission abatement strategies on air quality. Source apportionment, however, continues to be used as a general support to air quality planning, regardless of the approach followed. The main motivations of this work are therefore to focus on the specific use of source apportionment in the context of air quality planning and to support the conclusions of Clappier et al. (2017) with real-world examples.

In the context of FAIRMODE, the “Forum for Air Quality Modelling in Europe” (http://fairmode.jrc.ec.europa.eu), an inter-comparison exercise was organized to compare different source apportionment approaches on a common dataset (Belis et al., 2019). A series of models using different methods were run over the region of Lens, France, and results inter-compared. The exercise highlighted important differences among the approaches and provided the elements for an initial investigation of the causes behind these differences. However, the fact that different methods were implemented in different Air Quality Models (AQM) did not allow to quantify precisely the difference caused by the source apportionment method alone versus the one related to the AQM uncertainty.

To correct for this, the current study uses both theoretical examples and real-world datasets to highlight and explain the differences among source apportionment methods. We explore the potential inconsistencies among the approaches, quantify the differences and discuss the implications that these differences may have on air quality planning. The purpose of this work is to explain why the most currently used source apportionment methods aiming to support air quality planning, deliver substantially different answers and provide recommendations on what method to use under different conditions.

## 2 Which approaches to source apportionment?

In the following, we use a broad definition of source apportionment to reflect the variety of usages currently covered by this discipline. Source-apportionment methods aim to determine the role of a given source to air pollution levels. The most frequently used source-apportionment methods can be classified in the following three categories:

(1) *Emission Reduction Impacts methods* (ERI) provide source *impacts* by differencing two AQM simulations performed with the full emission source and a reduced emission source. This method is also referred to as brute-force, sensitivity analysis or as the perturbation method. In this work, we differentiate ERI-LOW, *source allocation* (Thunis et al., 2018), in which emission sources are reduced by a limited amount to preserve linearity between emission and concentration changes, from ERI-HIGH in which AQM simulations are performed with larger emission reductions. The ERI-LOW approach is used in the GAINS (Amann et al., 2011) and FASST (Van Dingenen et al., 2018) modelling systems as well as in the SHERPA approach (Thunis et al., 2016; Clappier et al., 2015; Pisoni et al., 2017; Thunis et al., 2018). Most ERI-HIGH studies use the particular zero-out approach where sources are reduced by 100% (Osada et al., 2009; Huang et al., 2018; Wang et al., 2014; Wang et al., 2015). Approaches based on emission reduction impacts are widely used for source apportionment.

(2) *Mass-Transfer methods* (MT) are designed to estimate *contributions* as the mass of a pollutant transferred from the emission sources to the ambient concentrations. These include receptor-oriented models (MT-RM) that apportion observed concentrations of pollutants at a given point in space to sources by using statistical analysis to match common chemical and physical characteristics between source and air pollution samples (Viana et al., 2008; Belis et al., 2013; Watson et al., 2008; Hopke, 2010). Mass-transfer models include also source-oriented models (MT-SM). These are based on AQM in which tagging/labelling techniques are implemented to keep track of the origin of air pollutants throughout a model simulation (Kranenburg et al., 2013). These approaches require all traditional AQM inputs to be available, in particular detailed emission inventories. Examples of MT-SM models are the particle source apportionment technology (PSAT) within the CAMx model (Yarwood et al., 2004; Wagstrom et al., 2008; Kwok et al., 2013; ENVIRON, 2014), the tagged species source apportionment algorithm (TSSA, ISAM) within CMAQ (Bhave et al., 2007; Wang et al., 2009) and the labeling module built in LOTOS-EUROS (Kranenburg et al., 2013).

(3) *Incremental methods* (INC) deliver *increments*, based on spatial gradients of concentration, calculated as the difference between concentrations at two specific locations (one influenced by the source, the other not). The incremental approach initially proposed by Lenschow et al. (2001) is used in many city air quality plans (Berlin, 2014; Segersson et al., 2017), in modelling studies (Squizzato and Masiol, 2015; Timmermans et al., 2017; Keuken et al., 2013; Ortiz and Friedrich, 2013; Pey et al., 2010) or in combined model-measurements analysis, to distinguish and quantify the street vs. the urban and/or the urban vs. the regional *contributions* (Kiesewetter et al., 2015).

**Fig. 1:**
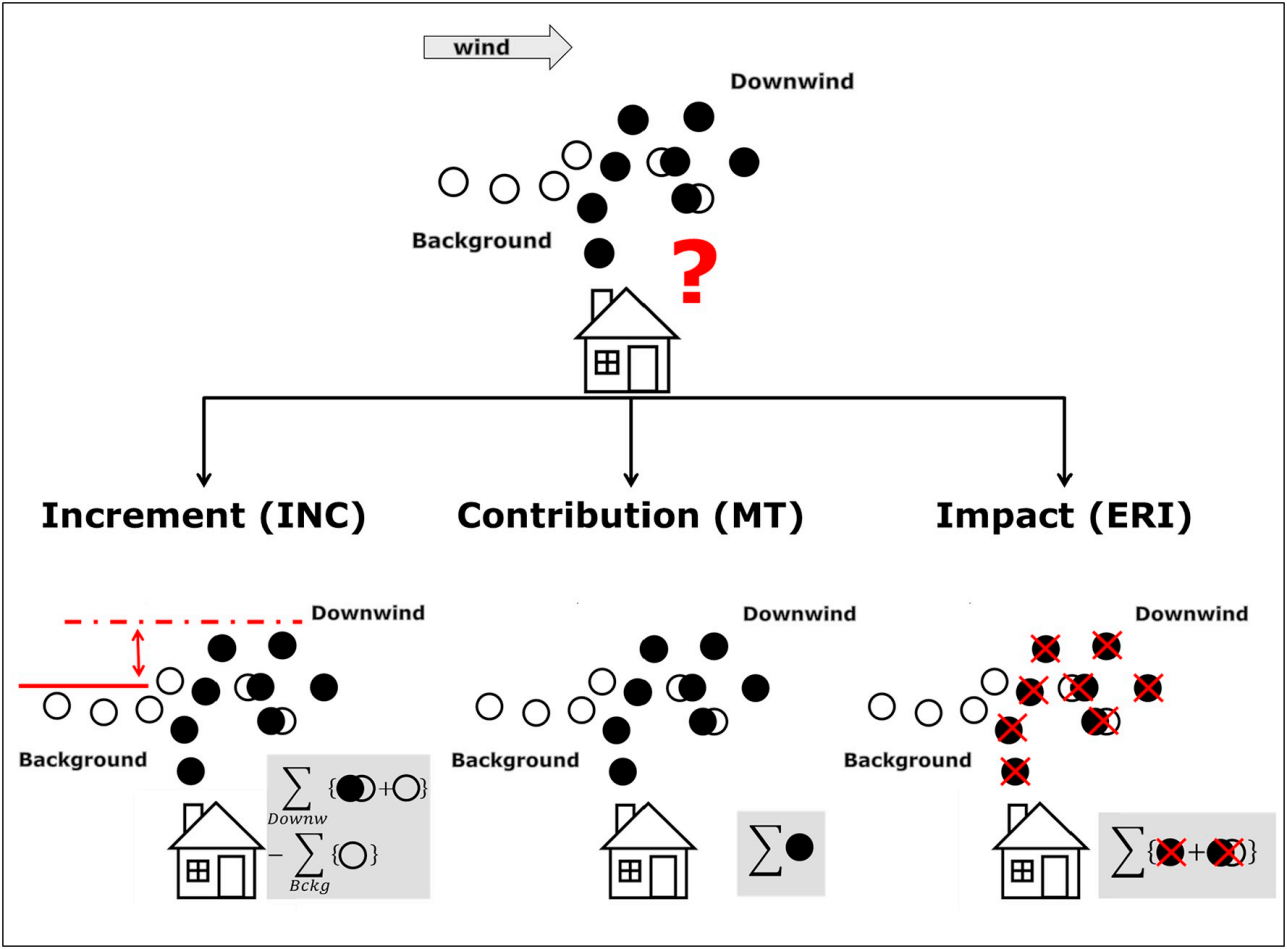
The main question addressed by source apportionment is; “What is the influence of a given emission source (e.g. residential heating from which emissions are schematically represented by black circles) to pollution levels at a given receptor location?” In this example, residential emissions (filled black circles) mix with the background pollution (empty circles) and generate secondary products (combined circles). *Increments* (lower left) are obtained by subtracting the background from the polluted downwind mass, *contributions* sum up the mass emitted by the source (sum of black circles) whereas *impacts* correspond to the change of mass resulting from the elimination of the source. The abbreviations “downw” and “bckg” stand for downwind and background.

The mechanism in which the three source apportionment approaches manage the calculation of the components is depicted schematically in Fig. 1, in a specific example to determine the importance of residential heating sources to pollution levels.

**Fig. 2:**
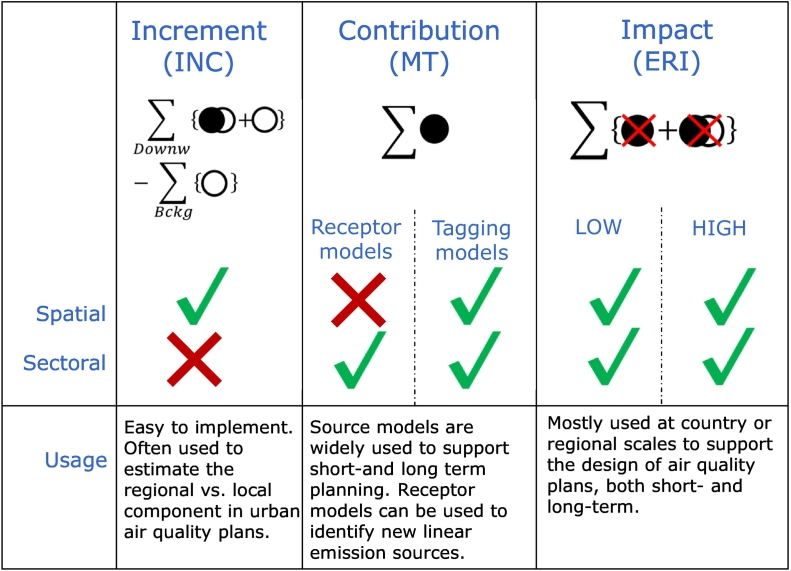
Main characteristics of the source apportionment methods discussed here, in terms of their ability to distinguish the spatial (e.g. urban vs. regional) and sectoral (e.g. transport vs. residential heating) origin of air pollution.

*Increments* (INC) are generally limited to the quantification of the spatial origins of pollution while receptor-modelling *contributions* (MT-RM) are limited to its sectoral origins, and to linear species (Kranenburg et al., 2013; Belis et al., 2013; Hendriks et al., 2013). By linear, we mean chemical species for which a linear relationship exists between a given change of the emission source and the resulting change in concentration at a receptor location. Examples of linear species include passive species that remain stable with time (e.g. primary particulate matter); species that undergo ageing processes (e.g. aged marine salt (Scerri et al., 2018)) or “linear” secondary species, as some secondary organic species (Srivastava et al., 2018; Wang et al., 2018; Zhao et al., 2018). Examples of non-linear species are species that are affected by second or higher order chemical reactions (e.g. ozone or secondary inorganic PM). In contrast, source oriented *contributions* (MT-SM) obtained with tagging/labelling modules and *impacts* (ERI) can be used to quantify either the spatial or sectoral origins of the pollution, or both (see Fig. 2).

It is worth noting that source apportionment applications often use methods in combination. The Berlin air quality plan (Berlin2014) distinguishes the urban from the regional components with *increments* while *contributions* are used as a follow-up step to identify and quantify the sectoral origins of the pollution. In the case of Stockholm (Segersson et al., 2017), *increments* and *impacts* are used in combination. Mertens et al. (2018) use *impacts* and *contributions* in complement, the first to assess the efficiency of mitigation measures on O_3_ levels and the second to retrieve additional information on unmitigated emission sources (i.e. those not covered by the *impacts*).

## 3 How do source apportionment approaches compare?

All source apportionment approaches deliver components (*increments*, *contribution* or *impacts*) assigned to different (spatial/sectoral) sources. In order to support air quality plans efficiently, i.e. to assess the efficiency of mitigation measures, these components need to be:

• **Dynamic**: components reflect the influence of emission changes on concentration.
• **Unambiguous**: components relate explicitly to one and only one source or one group of sources
• **Additive**: The sum of the components estimated for each source individually is equal to the component estimated for all sources at once.

In the following, we focus our attention on how the components obtained with different source apportionment methods fulfill these three criteria. Because the results of different source-apportionment methods vary when we apply them for linear or non-linear chemical species (because of their intrinsic assumptions), we distinguish linear from non-linear species in our analysis. Both theoretical and real-world examples are used in this work. While the theoretical examples highlight differences and issues, the real-world datasets (AQM simulations performed over the Po-valley region (Italy)) serve to quantify these differences.

### 3.1 Description of the theoretical examples

**Fig. 3:**
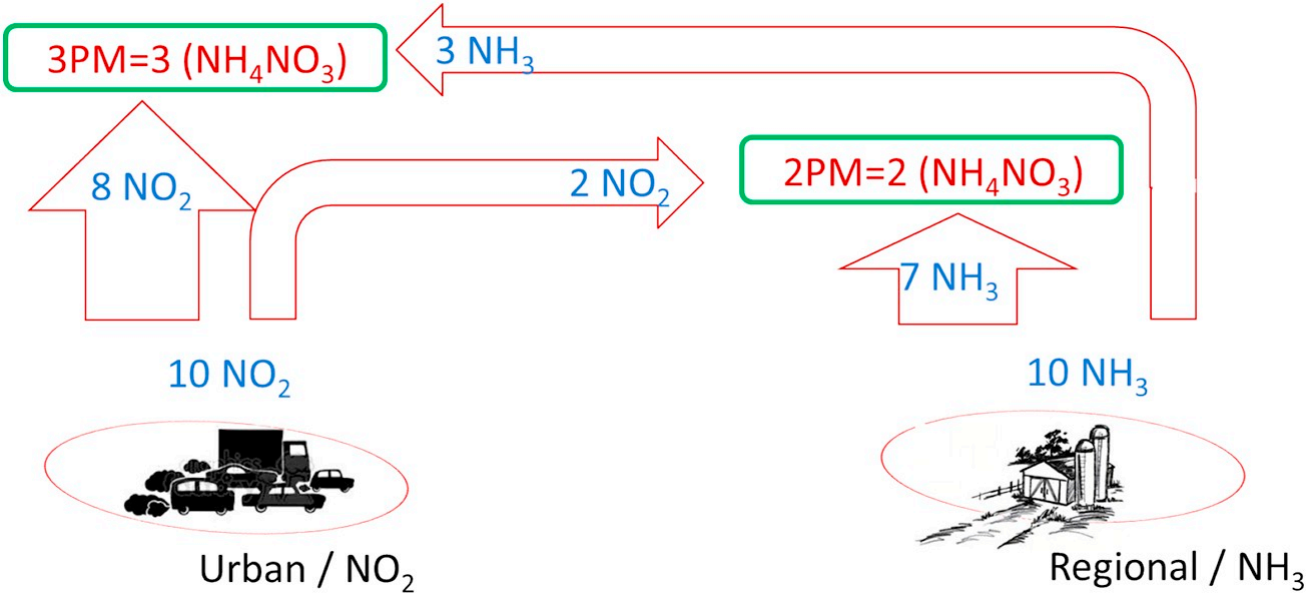
Theoretical example illustrating a simplified version of the formation mechanisms of secondary particulate matter (limited to NH_4_NO_3_) from emissions originating from two specific sectors of activity and geographical areas. See additional information in the text.

To highlight the differences in terms of source-apportionment approach, we use here simplified versions of real word processes with different levels of complexity. We first focus on the formation of particulate matter (PM) limited to ammonium nitrate (NO_3_NH_4_) originating from the reaction between nitrogen oxides (NO_2_) and ammonia (NH_3_). Our example is restricted to two emission sources: NO_2_ emissions in an urban area and NH_3_ emissions in a (nearby) regional area. For the formation of PM one molecule of NO_2_ and NH_3_ is required. Therefore, both the amount of NO_2_ and that of NH_3_ can be limiting the formation of PM (see Fig. 3). We further assume that no background pollution is present. In other words, there is no PM or gas-phase PM precursors present in the atmosphere when emissions from these two pollutants in these two areas are set to zero. The initial conditions are assumed to correspond to an availability of 10 mol both for NO_2_ and NH_3_ and we assume that two of the NO_2_ moles reach the regional area while three of the NH_3_ moles reach the urban area. Note that the reasoning remains valid for other choices of initial assumptions. Results obtained for this simplified situation are reported in Fig. 6.

**Fig. 4:**
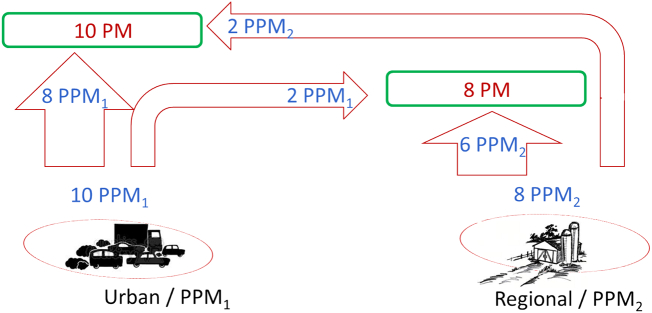
Theoretical example illustrating a simplified version of the formation mechanisms of primary particulate matter from emissions originating from two specific sectors of activity and geographical areas. See details in text.

In a second example, we design a similar theoretical example to compare source-apportionment approaches, but limited to chemically linear species, such as primary PM (PPM). We consider two types of PPM (denoted as PPM_1_ and PPM_2_) emitted by the two sources as presented in Fig. 4. For convenience, we assume that PPM_1_ and PPM_2_ have similar molar weights. This choice has no influence on the results and their implications and all derivations can easily be repeated for different split of molar weights.

**Fig. 5:**
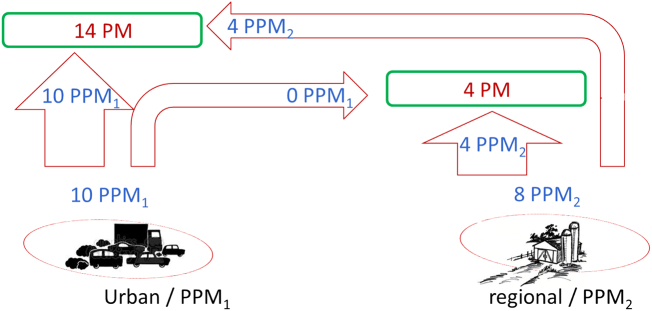
Theoretical example illustrating a simplified version of the formation mechanisms of primary particulate matter from emissions originating from two specific sectors of activity and geographical areas. Emissions are such that (1) urban emissions do not influence the rural area and (2) the rural area emissions lead to concentrations that are homogeneously distributed over the entire area (equal background).

Finally, we consider a third case with different urban to rural (and vice-versa) pollutant flows (Fig. 5). Primary PM is again formed by two types of PPM (PPM_urb_ and PPM_reg_ with similar molecular weights) being emitted by the two sources, but we assume in addition that (1) the urban emissions (PPM_urb_) do not influence the rural area and that (2) the rural area emissions lead to concentrations that are homogeneously distributed over the entire area [i.e. a same quantity of PPM_reg_ at the urban and rural locations (equal background)].

All calculations presented below are performed on the first test-case for convenience but a similar reasoning applies to the other cases.

### 3.2 Impacts (ERI)

The ERI approach consists in performing AQM simulations in which emissions from a given sector/area are reduced by a percentage (α) and calculating the resulting change of concentration. The *impact* of a source can be estimated by switching off emissions entirely (α =100%) for a given sector/area or by reducing emissions by a smaller amount and scale the concentration change to 100% (e.g. multiply by five the concentration change resulting from a 20% emission reduction, assuming a linear behaviour). An *impact* based on α = 20% is then representative of moderate emission reductions (i.e. close to the baseline levels) while at α = 100% the *impact* is representative of a complete activity switch-off. If chemical processes are non-linear for a given species, the two *impacts* will differ.

According to the above, the relative urban/NO_2_ *impact* (UIM) at a given location “l” is defined as:

$${\left[{\mathrm{UIM}}_{\alpha }^{l}\right]}_{\%}=\frac{{∆\mathrm{PM}}_{U\left(\alpha \right)}^{l}}{\alpha {\mathrm{PM}}^{l}}$$

where *∆PM*_*U*(*α*)_^*l*^ is the PM concentration change resulting from a reduction of the urban/NO_2_ emissions (U) by a percentage α and *PM*^*l*^ is the baseline PM concentration at that location.

A similar expression can be defined for the regional/NH_3_ *impact* (RIM).

$${\left[{\mathrm{RIM}}_{\alpha }^{l}\right]}_{\%}=\frac{{∆\mathrm{PM}}_{R\left(\alpha \right)}^{l}}{\alpha {\mathrm{PM}}^{l}}$$

**Fig. 6:**
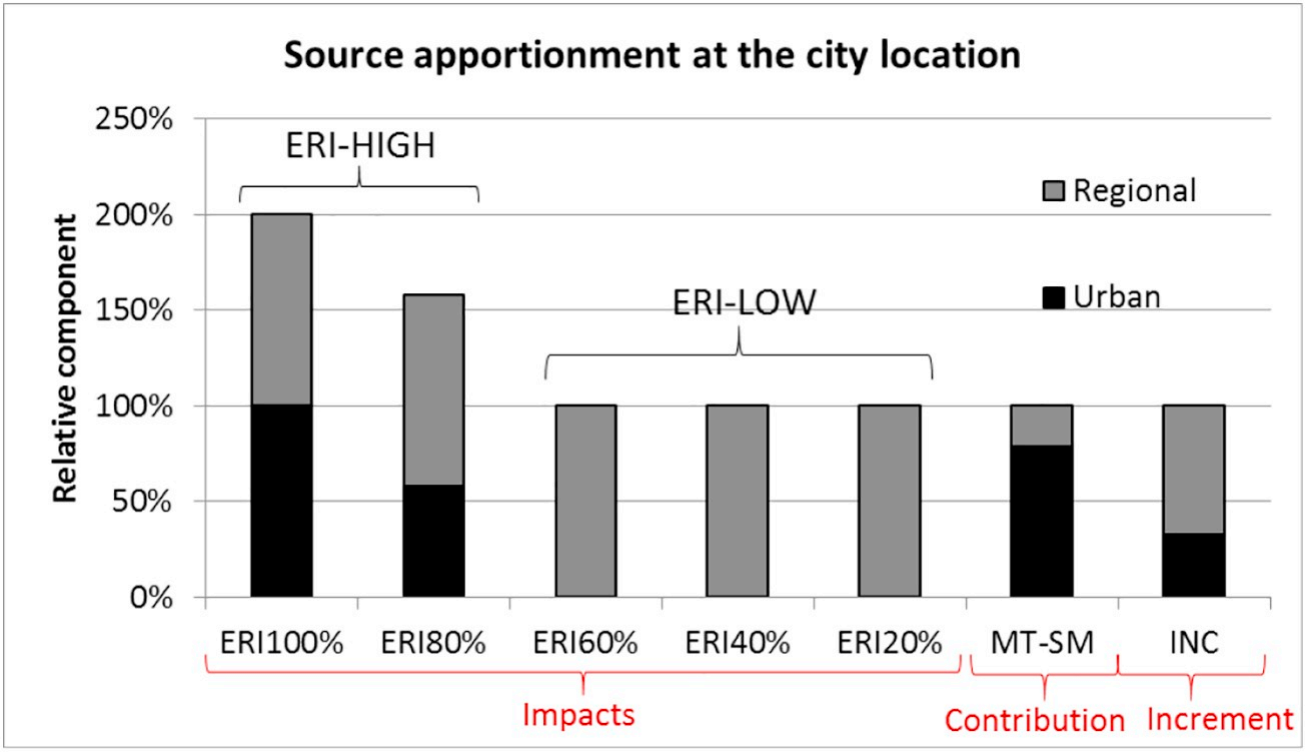
Overview of the urban/NO_2_ and regional/NH_3_*impacts*, *contributions* and *increments* to PM (limited to ammonium nitrate) at the city location obtained for example 1 with the emission reduction *impacts* (ERI) approach for different percentage reduction levels, with the Mass Transfer (MT-SM) and incremental (INC) approaches, respectively. *Impacts* are split into source allocation (ERI-LOW) and ERI-HIGH, according to the level of emission reduction (see text for details).

The five most left columns in Fig. 6 report the urban/NO_2_ and regional/NH_3_ *impacts* to PM obtained with different levels of emission reductions for the simplified case presented in Fig. 3 at the city location “l”. It is interesting to note that while the regional/NH_3_ impacts are linear (i.e. ${∆\mathrm{PM}}_{R\left(\alpha_{1}\right)}^{l}/\alpha_{1}{\mathrm{PM}}^{l}={∆\mathrm{PM}}_{R\left(\alpha_{2}\right)}^{l}/\alpha_{2}{\mathrm{PM}}^{l}\Rightarrow {∆\mathrm{PM}}_{R\left(\alpha_{2}\right)}^{l}=\frac{\alpha_{2}}{\alpha_{1}}{∆\mathrm{PM}}_{R\left(\alpha_{1}\right)}^{l}$), this is not the case for the urban/NO_2_ *impacts*. Indeed, these *impacts* remain null for percentage reductions up to about 60% and start growing beyond that value. This is a consequence of the NH_3_ limited regime in the city location where NO_2_ concentrations are in excess, compared to the availability of NH_3_. Limited NO_2_ emission reductions then remain inefficient and do not change the PM concentration. However, when NO_2_ emission reductions are larger than 60%, the chemical regime changes from NH_3_ to NO_2_ limited and the urban/NO_2_ *impacts* start growing. On the other hand, the regional/NH_3_ *impacts* show a linear behaviour over the entire range of reductions because the PM concentrations continue to be determined by NH_3_ levels regardless of the intensity of the NH_3_ emission reductions.

While Burr and Zhang (2011b) noted that *impacts* are valuable for policy makers to analyse the effects of emission reductions on air quality because of their inherent simplicity, they also flag out the issue that the sum of all source *impacts* does not always equal the baseline concentrations. In other words, the *impacts* obtained from each source category are not always additive.” If we translate this to our simple example, as shown in Fig. 6, the sum of the urban/NO_2_ and regional/NH_3_ *impacts* is equal to 200% of the PM baseline concentrations for α = 100% and to 160% for α = 80% while it is equal to 100% for all reductions levels (α) below 60%. As shown by Stein and Alpert (1993) and Clappier et al. (2017), the sum of the individual source *impacts* must be complemented with non-linear interaction terms to ensure that the sum of all terms equals the PM baseline concentration. In the case of our two sources example, the total concentration at location “l” (*PM*^*l*^) is expressed as the sum of the two individual concentration changes (by a percentage α, i.e. *∆PM*_*R*(*α*)_^*l*^ and *∆PM*_*U*(*α*)_^*l*^), one non-linear interaction term (${\hat{\mathrm{PM}}}_{\alpha }^{l})$ and a term that represents the concentration remaining when both sources are reduced (*PM*_*UR*(*α*)_^*l*^):

(1) $${\mathrm{PM}}^{l}={\mathrm{PM}}_{\mathrm{UR}\left(\alpha \right)}^{l}+∆{\mathrm{PM}}_{U\left(\alpha \right)}^{l}+{∆\mathrm{PM}}_{R\left(\alpha \right)}^{l}+{\hat{\mathrm{PM}}}_{\mathrm{UR}\left(\alpha \right)}^{l}$$

While each individual reduction terms is unambiguously related to one source, this is not the case of the interaction term that can therefore not be apportioned. ERI-HIGH and ERI-LOW differ in the way they deal with this non-linear interaction term.

#### 3.2.1 Source allocation (ERI-LOW)

In our example, the interaction terms remain null for emission reductions less than about 60%, a level of NO_2_ reduction at which the chemical regime changes from NH_3_ to NO_2_ limited, implying non-linearities in the relation between emission and concentration changes. We refer the ERI based source apportionment as source allocation (Thunis et al., 2018) over the range of emission reductions where interaction terms are negligible (ERI-LOW). We denote by α_t_ its threshold level of application, the level at which the emission reductions prescribed in the method are small enough to represent the same chemical regime as in the situation under consideration (here around 60%).

With ERI-LOW, the PM concentration is decomposed in a sum of unambiguous *impacts* (urban (UIM) and regional (RIM)), equal to the *impacts* calculated with emission reductions falling within the linear range of validity (*α* ≤ *α*_*t*_). A residual component (RE) is obtained by difference.

(2) $${\mathrm{PM}}^{l}={\mathrm{RE}}_{\mathrm{ERI}-\mathrm{LOW}}^{l}+{\mathrm{UIM}}_{\mathrm{ERI}-\mathrm{LOW}}^{l}+{\mathrm{RIM}}_{\mathrm{ERI}-\mathrm{LOW}}^{l},$$

where

$$\begin{array}{l} {\mathrm{UIM}}_{\mathrm{ERI}-\mathrm{LOW}}^{l}=\frac{\Delta {\mathrm{PM}}_{U\left(\alpha \right)}^{l}}{\alpha };{\mathrm{RIM}}_{\mathrm{ERI}-\mathrm{LOW}}^{l}=\frac{\Delta {\mathrm{PM}}_{R\left(\alpha \right)}^{l}}{\alpha }\text{and}\\ {\mathrm{RE}}_{\mathrm{ERI}-\mathrm{LOW}}^{l}={\mathrm{PM}}^{l}-\frac{\Delta {\mathrm{PM}}_{U\left(\alpha \right)}^{l}}{\alpha }-\frac{\Delta {\mathrm{PM}}_{R\left(\alpha \right)}^{l}}{\alpha }\text{with}\;\alpha \leq \alpha_{t}, \end{array}$$

In relative values (percentage of the baseline PM concentration), these *impacts* are expressed as:

$${\left[{\mathrm{UIM}}_{\mathrm{ERI}-\mathrm{LOW}}^{l}\right]}_{\%}=\frac{{∆\mathrm{PM}}_{U\left(\alpha \right)}^{l}}{\alpha {\mathrm{PM}}^{\left(l\right)}}\;{\left[{\mathrm{RIM}}_{\mathrm{ERI}-\mathrm{LOW}}^{l}\right]}_{\%}=\frac{{∆\mathrm{PM}}_{R\left(\alpha \right)}^{l}}{\alpha {\mathrm{PM}}^{l}}$$

$$\;{\left[{\mathrm{RE}}_{\mathrm{ERI}-\mathrm{LOW}}^{l}\right]}_{\%}=1-{\left[{\mathrm{UIM}}_{\mathrm{ERI}-\mathrm{LOW}}^{l}\right]}_{\%}-{\left[{\mathrm{RIM}}_{\mathrm{ERI}-\mathrm{LOW}}^{(l}\right]}_{\%}$$

It is worth noting that estimating the threshold level of application (α_t_) is not straightforward and requires additional efforts in terms of AQM simulations. This particular issue is discussed in the real world examples of Section 4.3.

Let's test this formulation with our simple example. For α = 25%, *∆PM*_*U*(25%)_^*l*^ is estimated by reducing the emissions available from the urban area by 25%, i.e. from 8 to 6 mol that react with the 3 mol of NH_3_. The combination of 6 mol of NO_2_ with 3 mol of NH_3_ leads to 3 mol of PM, implying that *∆PM*_*U*(25%)_^*l*^ = 0. Therefore,*UIM*_*ERI*−*LOW*_^*l*^ = 0. The same process can be repeated for other reduction levels and will lead to similar results (*UIM*_*ERI*−*LOW*_^*l*^ = 0) as long as relation (2) is fulfilled, i.e. for reductions below about 60% in our example. This level represents the application threshold (*α*_*t*_) for the source allocation approach in this example.

#### 3.2.2 ERI-HIGH

ERI-HIGH refers to the application of ERI beyond the threshold level *α*_*t*_. Similarly to ERI-LOW, the *impacts* still reflect the effect of emission changes on concentrations. However, because of non-linear interaction terms, *impacts* are not anymore additive, i.e. the *impact* of a simultaneous reduction of two sources differs from the sum of their individual *impacts*. From Eq. (1):

$${\mathrm{PM}}^{l}-{\mathrm{PM}}_{\mathrm{UR}\left(\alpha \right)}^{l}={∆\mathrm{PM}}_{\mathrm{UR}\left(\alpha \right)}^{l}\neq {∆\mathrm{PM}}_{U\left(\alpha \right)}^{l}+{∆\mathrm{PM}}_{R\left(\alpha \right)}^{l}$$

In our example of Fig. 3 with α = 80%, *PM*_*UR*(80%)_^*l*^ = 2.4 ≠ *∆PM*_*U*(80%)_^*l*^ + *∆PM*_*R*(80%)_^*l*^ = 1.4 + 2.4 = 3.8. This implies that ${\left[{\mathrm{UIM}}_{\mathrm{ERI}-\mathrm{HIGH}}^{l}\right]}_{\%}+{\left[{\mathrm{RIM}}_{\mathrm{ERI}-\mathrm{HIGH}}^{l}\right]}_{\%}=\frac{1.4}{3*0.8}+\frac{2.4}{3*0.8}=0.58+1\neq 1$.

The only option to recover additivity would be to account for all non-linear interaction terms (e.g. ${\hat{\mathrm{PM}}}_{\mathrm{UR}\left(\alpha \right)}^{l}$ in Eq. (1)). However, because these terms represent interactions between sources, they cannot be attributed unambiguously to one source, hence preventing a proper source apportionment.

In addition, *impacts* are valid only for the specific emission reductions on which they are constructed, implying a lack of robustness. In other words, nothing guarantees that an *impact* obtained at *α* = 75% remain valid at 90%. In our example: [*UIM*_*ERI*−*HIGH*_^*l*^]_%_ = 1 at α = 100% but 0.58 at α = 80% and 0 below α = 60%.

### 3.3 Contributions (MT)

This source-apportionment approach decomposes the pollutant mass into a sum of *contributions*, each associated to a given emission precursor.

$${\mathrm{PM}}^{l}={\mathrm{RE}}^{l}+{\mathrm{UC}}^{l}+{\mathrm{RC}}^{l}$$

where *UC*^*l*^ and *RC*^*l*^ are the urban and regional *contributions* and *RE*^*l*^ a residual *contribution* that represents the PM fraction resulting from all emission sources other than NO_2_/urban and NH_3_/regional, such as, for instance, the background. In our example, the residual *contribution* is zero.

In our example, the mass of PM (considered as NO_3_NH_4_) is split into a mass of NO_3_, attributed to an urban/NO_2_ origin and into a mass of NH_4_, attributed to a regional/NH_3_ origin. At the city location, the urban/NO_2_ *contribution* (only MT-SM as receptor models are limited to linear species) is defined as the ratio between the molar mass of NO_3_ and the molar mass of the total PM concentration:

$${\left[{\mathrm{UC}}_{\mathrm{MT}-\mathrm{SM}}^{l}\right]}_{\%}=\frac{3M_{{\mathrm{NO}}_{3}}}{3\left(M_{{\mathrm{NO}}_{3}}+M_{{\mathrm{NH}}_{4}}\right)}=\frac{62}{80}=77.5\%$$

while the regional/NH_3_ *contribution* (RC) is, by construction, complementary and equal to

$${\left[{\mathrm{RC}}_{\mathrm{MT}-\mathrm{SM}}^{l}\right]}_{\%}=\frac{3M_{{\mathrm{NH}}_{4}}}{3\left(M_{{\mathrm{NO}}_{3}}+M_{{\mathrm{NH}}_{4}}\right)}=22.5\%$$

It is important to note that in this decomposition, the final contributions depend (1) on the choice of the chemical pathway to track a precursor to its corresponding secondary compound (here: NO_2_ - > HNO_3_ - > NO_3_ and NH_3_ ➔ NH_4_) but also (2) on the relative weights chosen to split the secondary compounds. In this example, the NH_4_ and NO_3_ are attributed according to their molecular weights but other options (e.g. mole) are possible (e.g. Kranenburg et al., 2013). The second column from the right in Fig. 6 shows these results for the MT-SM *contribution*.

### 3.4 Increments (INC)

With the incremental approach, the PM concentration at a city location is divided in two components as follows:

$${\mathrm{PM}}^{l}=\underset{{\mathrm{UI}}^{l}}{\underbrace{\left[{\mathrm{PM}}^{l}-{\mathrm{PM}}^{l_{0}}\right]}}+\underset{{\mathrm{RC}}^{l}}{\underbrace{{\mathrm{PM}}^{l_{0}}}}$$

in which the urban *increment* (UI), is estimated as the concentration spatial difference between two sites: rural (l_0_) and city background (l), regardless of the atmospheric and chemical processes that generated the concentration gradient. From the example presented in Fig. 3 the incremental approach leads to a relative urban *increment* at location (l), equal to:

$${\left[{\mathrm{UI}}^{l}\right]}_{\%}=\frac{{\mathrm{PM}}^{l}-{\mathrm{PM}}^{l_{0}}}{{\mathrm{PM}}^{l}}=\frac{\left(3-2\right)}{3}=33\%$$

The regional/NH_3_ *increment* at the city location is by construction complementary and equal to ${\left[{\mathrm{RI}}^{l}\right]}_{\%}=\frac{{\mathrm{PM}}^{l_{0}}}{{\mathrm{PM}}^{l}}=\frac{2}{3}=67\%$. Similarly to *contributions* (MT), *increments* are additive by construction and represent the apportionment of 100% of the emissions.

Thunis (2018) recently showed that the urban *increment* (*PM*^*l*^ − *PM*^*l*_0_^) was related to the *impact* (*∆PM*_*U*(100%)_^*l*^) through the following relation:

$$\Delta {\mathrm{PM}}_{U\left(100\%\right)}^{l}=\underset{{\mathrm{UI}}^{l}}{\underbrace{\left({\mathrm{PM}}^{l}-{\mathrm{PM}}^{l_{0}}\right)}}+\underset{\mathrm{City spread}}{\underbrace{\Delta {\mathrm{PM}}_{U\left(100\%\right)}^{l_{0}}}}+\underset{\mathrm{Background deviation}}{\underbrace{\left({\mathrm{PM}}_{U\left(100\%\right)}^{l}-{\mathrm{PM}}_{U\left(100\%\right)}^{l_{0}}\right)}}$$

As indicated by this relation, the *increment* accurately represents an *impact* only if both of the following two assumptions are fulfilled:

(1) Zero city spread: the urban emissions have no influence at the rural location (∆PM_U(100%)_^*l*_0_^ = 0), and

(2) Equal background: the background (concentration obtained when the city is switched off) is identical at the rural and city locations (PM_U(100%)_^*l*^ − PM_U(100%)_^*l*_0_^ = 0).

These two assumptions imply contradictory constraints on the choice of the two locations and are therefore challenging to meet. For the example under consideration, neither assumption is fulfilled.

### 3.5 Comparative overview

The differences between the three approaches are a direct outcome of the underlying methodological assumptions, as summarized below.

(1) *Increments (INC):* *Increments*, e.g. regional and urban, result from difference between levels of concentrations and are *additive* by construction. Thunis (2018) showed, however, that these components are unambiguously associated to the sources only if two assumptions are fulfilled: (1) urban emissions must not influence the regional location and (2) the background (levels reached when urban emissions are switched off) must be equal at both the urban and regional locations. When these two assumptions are not met, *increments* become *ambiguous* as they both include a mix of urban and regional influences. This ambiguity implies that *increments* are not *dynamic* because they do not reflect concentration changes resulting from emission changes. Only when the two assumptions are fulfilled, *increments* become dynamic but they then only reflect the *impact* of a full emission change (100%) and cannot be extrapolated to any other emission change because of non-linearities.

(2) *Contributions (MT-SM)*: *Contributions* are based on the estimation of the mass fraction derived from the tagged precursors. They are *additive* and *unambiguous* by construction. This unambiguity is however obtained at the expense of the neglect of indirect chemical effects. In other words, each species in tagging/labelling approach is linked only to its direct primary precursor (e.g., NO_2_ ➔ NO_3_, NH_3_ ➔ NH_4_) (i.e., direct effect) and the effect of non–direct emission precursors (e.g., NH_3_ emissions can affect the formation of NO_3_) is not considered (Burr and Zhang, 2011b; Pun et al., 2008). In our example, the urban *contribution* is based on NO_3_ mass only. Because of this neglect of indirect effects, *contributions* are not *dynamic*. This was confirmed by Burr and Zhang (2011b) who noted that the omission of such indirect effects in the current formulation of tagging/labelling source apportionment approaches limit their use to support the planning of secondary PM species. Along the same line, Qiao et al. (2018), Grewe et al. (2010, 2012), Clappier et al. (2017) and Mertens et al. (2018) conclude that tagging approaches are not designed to assess the consequences of emission changes on air quality.

(3) *Impacts (ERI)*: *Impacts* are obtained by reducing emissions from the different precursors by a given percentage (α). In contrast with *contributions*, *impacts* account for indirect chemical effects that appear when emission are reduced. They are therefore *dynamic* and *unambiguous* by construction. Nevertheless, these properties are obtained at the expense of a lack of additivity (i.e. the *impact* of a combined reduction of precursors does not equal the sum of the precursor individual *impacts*). ERI-HIGH *impacts* then vary with the reduction percentage and are not additive. However, over a range of moderate enough values of α (source allocation, ERI-LOW), *impacts* remain *additive* and constant, implying that their validity extends over this limited range of emission reductions.

Fig. 6 clearly illustrates that the *impacts* (ERI) vary with the precursor emission reduction percentage. The urban *impact* equals 100% for α = 100% while it equals 0% at α = 60%. Below that threshold, the *impacts* remain constant. In contrast to *impacts*, *contributions* and *increments* lead to a single estimate, therefore independent from the emission reduction percentages. And this *contribution* or *increment* single value is very different from the *impacts* estimates. The urban *increment* reaches 30% while the urban *contribution* is 77.5%. These differences are large and can lead to very different air quality plan designs, some of them leading to non-effective actions.

**Fig. 7:**
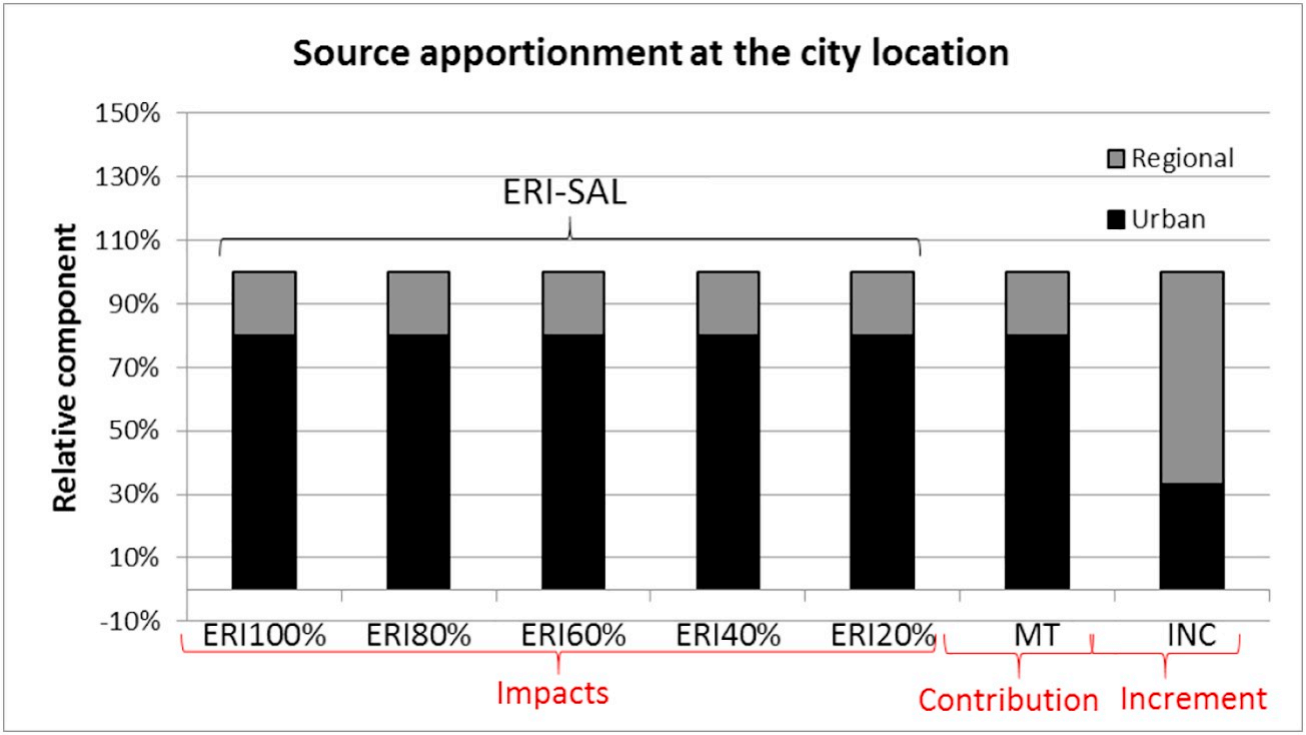
Overview of the urban/PPM and regional/PPM *impacts*, *contributions* and *increments* at the city location for PM (limited to PPM) obtained with the ERI approach for different percentage reduction levels, with the Mass Transfer (MT) and incremental (INC) approaches, respectively. Because of linearity, source allocation (ERI-LOW) can be applied up to emission reductions of 100% and the ERI-HIGH does not appear. See text for details.

For linear species (Fig. 7), both the *contributions* (MT-RM and MT-SM) and *impacts* provide similar responses over the entire range of emission. Because responses are linear over the whole range, source allocation (ERI-LOW) is applicable everywhere and ERI-HIGH is not relevant. Both *contributions* and *impacts* meet the three criteria (unambiguous, dynamic and additive). On the other hand, *increments* do not, for the same reasons as detailed under the non-linear case.

**Fig. 8:**
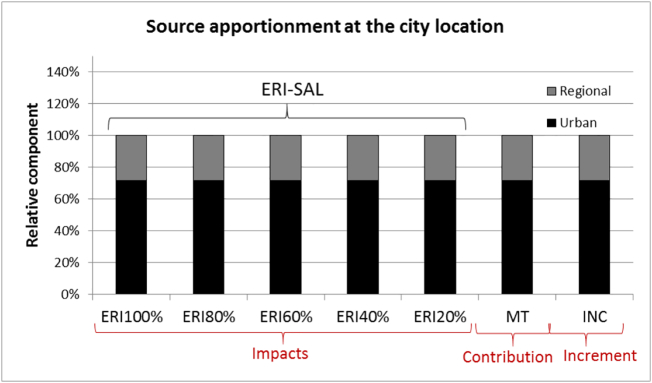
Overview of the urban/PPM and regional/PPM components at the city location for PM (limited to PPM) obtained with the ERI approach for different percentage reduction levels, and with the Mass Transfer (MT) and incremental (INC) approaches. Emissions are imposed to fulfill the two incremental assumptions (zero city spread and equal background). See text for details.

When the two incremental assumptions are fulfilled (zero city spread and equal background), *contributions*, *impacts* and *increments* lead to identical results as indicated in Fig. 8.

These theoretical conclusions are supported by the real-world results as discussed below.

## 4 Real-world datasets

### 4.1 Impacts (ERI) vs. increments (INC)

Thunis (2018) recently assessed the validity of the two assumptions underpinning the incremental approach for a series of EU cities. While these assumptions are generally recognized, the extent of their fulfilment is never quantified. This is due in particular to the impossibility of performing this assessment via measurements. With a modelling approach based on SHERPA, this work showed that because of ambiguity between the components, the incremental approach was leading to an underestimation of the urban *impact* ranging from 30 to 50% for medium and large cities, with respect to a source allocation (ERI-LOW) approach.

When based on measurements, a major difficulty arises from the lack of representativeness of the monitoring stations and to the subsequent variability in terms of urban *increments* resulting from the choice of the station pairs. Thunis (2018) showed that depending on this choice, the *increment* could differ by a factor 2 in some cities. The fact that urban *increments* are very sensitive to distance and to the choice of the rural background station indicates the lack of robustness of this approach. It was concluded that the incremental approach, either based on measurement or modelling, is not suited to estimate *contributions* or *impacts* to air pollution.

### 4.2 Impacts (ERI) vs. contributions (MT-SM)

Differences between *contributions* and impacts have been discussed in many studies (Burr and Zhang, 2011a; Kranenburg et al., 2013; Clappier et al., 2017; Grewe et al., 2010; Mertens et al., 2018). We highlight these differences with results obtained with a real-world modelling dataset over the Po-Valley (Italy) where both *contributions* and impacts are calculated consistently. It consists of a series of AQM simulations performed with the CAMx model (ENVIRON, 2014) applied in ERI mode for different emission reduction scenarios. Three sectors of activity: agriculture, transport and industry are analysed and reduced by 50 and 100% over the Po-valley area (Italy). The area was selected because of the high levels of PM and considerable anthropogenic emissions that facilitate the analysis of the processes described in the theoretical examples. We refer to Pepe et al. (2016) for additional details on the base case model set-up. The three sectors: agriculture (A), transport (T) and industry (I) are reduced independently from each other and/or in a combined way. All possible combinations of reductions are performed leading to seven different simulations (A, T, I, AT, AI, TI, ATI, where A, T and I represent the agriculture, transport and industry, respectively) for each level of reduction, in addition to the baseline simulation. Based on the same set of input data, an additional simulation has been performed with CAMx using the built-in tagging species module (PSAT) to deliver *contributions* for the same three sectors with a MT-SM based approach. All simulations are performed with a resolution of 5 km for an entire meteorological year (2010 – reference year for the Italian emission inventory). All pollutant species are analysed at daily (PM) or hourly (O_3_, NO_2_…) frequency.

**Fig. 9:**
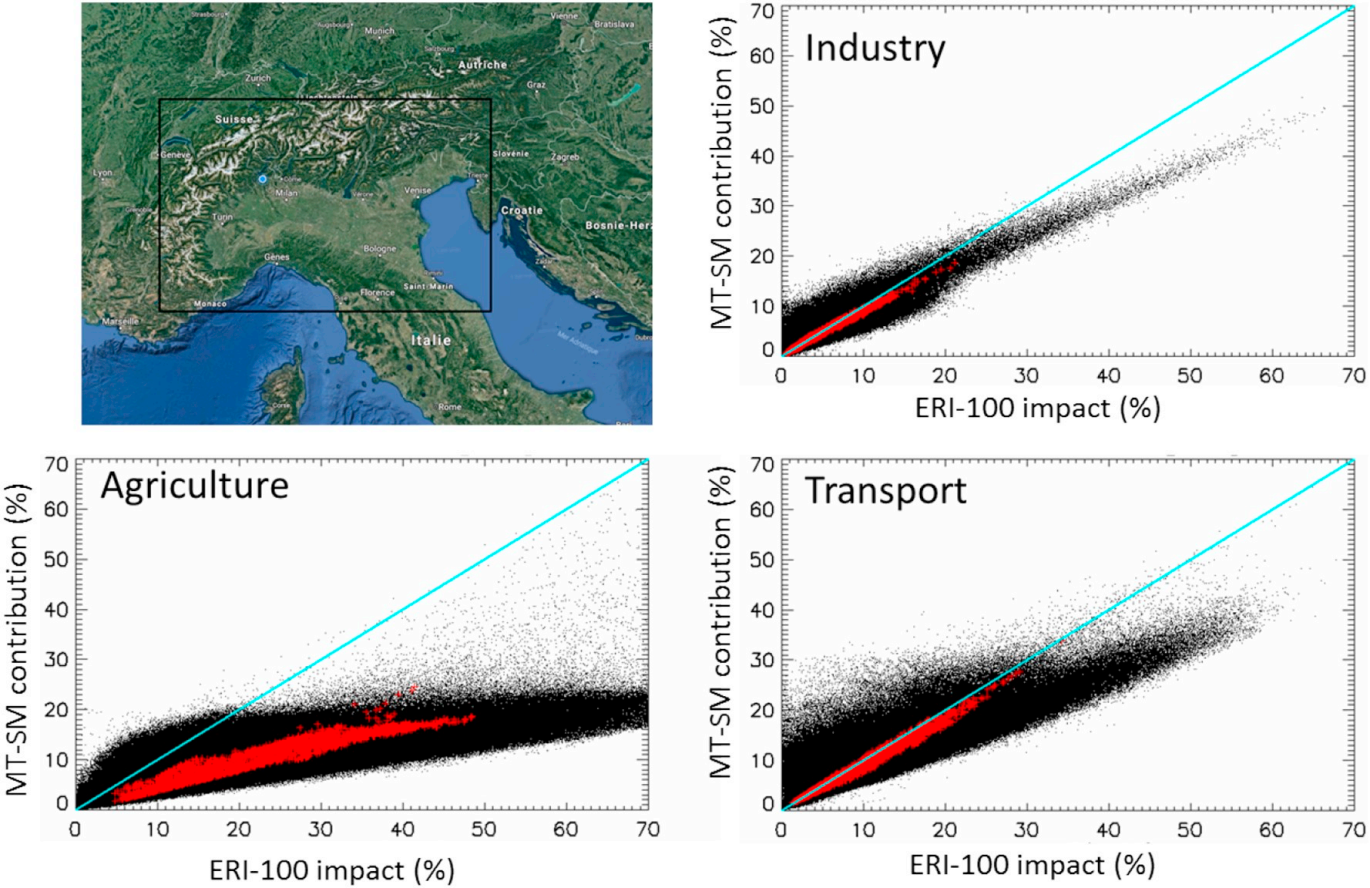
Comparison between relative *contributions* (MT-SM) and *impacts* (ERI) from the CAMx modelling study of the Po-Valley (shown top-left). Yearly averaged PM_2.5_ concentrations are shown in red while their corresponding 365 daily values are shown in black (number of points for daily average = number of grid cell domain locations * 365). (For interpretation of the references to colour in this figure legend, the reader is referred to the web version of this article.)

The ERI100% and ERI50% *impacts* are calculated as $\frac{{∆\mathrm{PM}}_{I\left(100\%\right)}^{\mathrm{Milan}}}{{\mathrm{PM}}^{\mathrm{Milan}}}$ and $\frac{2∆{\mathrm{PM}}_{I\left(50\%\right)}^{\mathrm{Milan}}}{{\mathrm{PM}}^{\mathrm{Milan}}}$, respectively (here the example is for industry with subscript I) while the MT-SM *contributions* are a direct output of PSAT. The comparison between *contributions* and *impacts* is made with ERI at 100% as this corresponds to the fraction apportioned by MT-SM (Kwok et al., 2013; Kranenburg et al., 2013; Burr and Zhang, 2011b) and the focus is on the dynamicity aspects for which ERI serves as a reference. Results for all grid-cell locations within the Po-valley modelling domain with a yearly PM_2.5_ average falling within the 80th highest percentile of the concentrations are selected for the analysis (Fig. 9). The underestimation of the agriculture *contribution* both for daily and yearly averages by MT-SM compared to the ERI *impact* is very large (up to a factor of three). *Contributions* and *impacts* from the transport and industry sectors agree quite well for yearly averages but show important differences for daily values (especially for industry), although not as substantial as for agriculture. The smaller differences observed for longer time averages can be explained by the reduced non-linearity effects with longer time averaging periods (Thunis et al., 2015). Designing air quality plans, based on a *contributions* (MT-SM), would therefore systematically underestimate the effect of reducing agricultural emissions and miss-quantify the consequences of other sectorial measures. These issues are more acute when dealing with short-term rather than long-term air quality plans.

Mertens et al. (2018) raised the point that although tagging *contributions* cannot assess and quantify the efficiency of mitigation measures on air quality, they are useful to assess the potential of additional unmitigated emission sources, i.e. the measures not covered by the ERI-LOW range. In other words, while the ERI-LOW *impacts* provide insight for single sector mitigation measures over a limited range of emission reductions (<α_t), tagging *contributions* provide information on the potential for combined/single measures beyond α_t. Tagging is therefore used as an additional information to support the interpretation of the ERI impacts. Because tagged *contributions* depend on arbitrary choices in terms of chemical pathways and in terms of splitting weights (molecular weight, mole…), this additional information remains however qualitative.

This real-world modelling dataset can also be used to define the boundary between the ERI-HIGH and ERI-LOW regimes, as discussed below.

### 4.3 Source allocation (ERI-LOW) vs. ERI HIGH

One of the key issues with ERI-LOW is to determine the threshold level (*α*_*t*_) that separates it from ERI-HIGH. Below this threshold, the following two conditions need to be fulfilled:

1. *Impacts* remain constant with the level of emission reduction, i.e. *ERI*(*α*_1_) = *ERI*(*α*_2_) for any *α*_1_ and *α*_2_ within the range [0, *α*_*t*_]

2. Given a specific α within the range [0, *α*_*t*_], non-linear interactions are negligible, implying additivity of the *impacts*: *ERI*_*U*_(α) + *ERI*_*R*_(α) ≅ *ERI*_*UR*_(α)

**Fig. 10:**
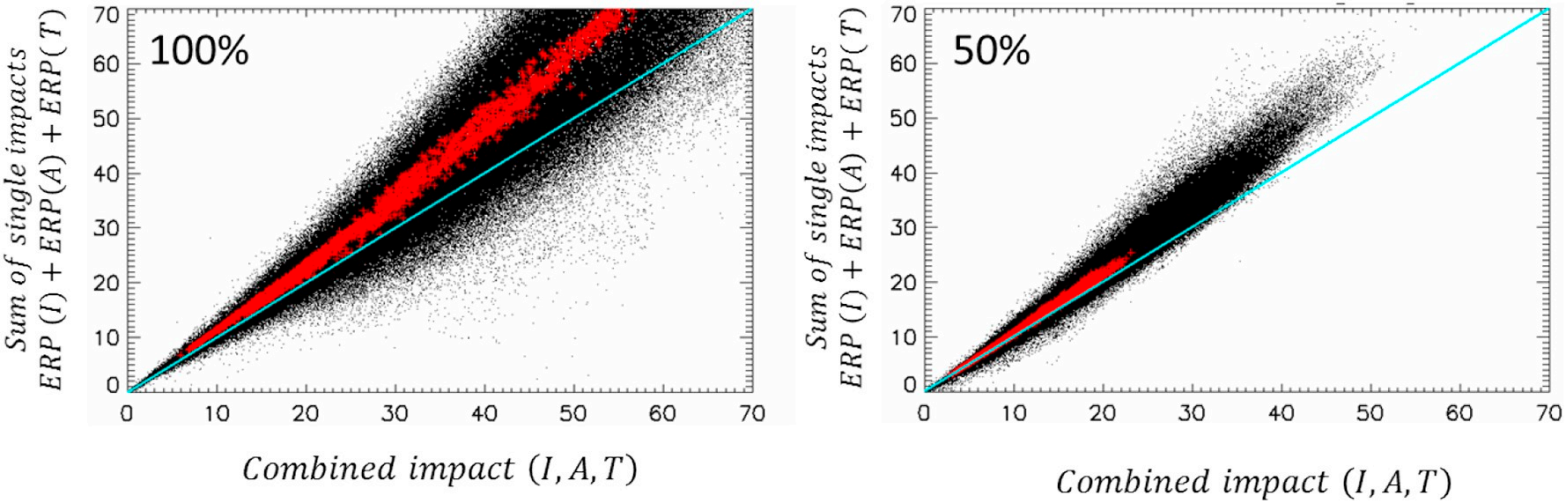
Comparison between combined *impacts* obtained when the three sources are reduced simultaneously (abscissa) and the sum of the individual *impacts* (ordinate) for 100% (left) and 50% (right) emission reductions. Yearly averages for all grid cells within the modelling domain are shown in red while the daily values (365 points for one yearly average) are shown in black. (For interpretation of the references to colour in this figure legend, the reader is referred to the web version of this article.)

The second condition can be tested by comparing the *impacts* of a simultaneous reduction of all three sectors (I, A and T) to the sum of the individual *impacts*. From Fig. 10, we see that this condition is fulfilled at 50% for yearly averages but not for all other options (i.e. neither at 50% for daily, nor at 100% for both yearly and daily). In order to determine more precisely the value of the threshold, this type of comparison should be repeated for different levels of emission reductions. Regarding the first condition, an additional simulation with a lower reduction (e.g. 25%) would be needed. As these are not available in the current datasets, we rely on Thunis et al. (2015) who performed these tests over the Po-valley with another AQM.

In theory, all tests should be performed each time a new version of the model (change in resolution, meteorology, emissions…) is available; in practice, this is quite demanding and tests are generally performed in a piecewise manner, i.e. some tests with a specific model version, others with another version, assuming that the basics of the physical and chemical behaviours of the atmosphere remain unchanged. For this dataset, α_t_ = 50% represents a valid upper limit for ERI-LOW for yearly or seasonal PM values. For other time averaging, α_t_ < 50%, and additional tests would be needed to determine precisely the threshold value.

## 5 When to use which method? Implications for policy

All source apportionment methodologies presented before are based on measurement or/and modelling data. As such, they are all affected by uncertainties (e.g. concerning the location of the measurement stations with the incremental approach or by the quality of the model and model input data for the mass-transfer or ERI approaches). While the accuracy of the apportioned components will improve with better quality data (measurement and/or modelling), it is important to stress that the discrepancies observed between *impacts*, *contributions* and *increment* will remain because they are different concepts.

**Fig. 11:**
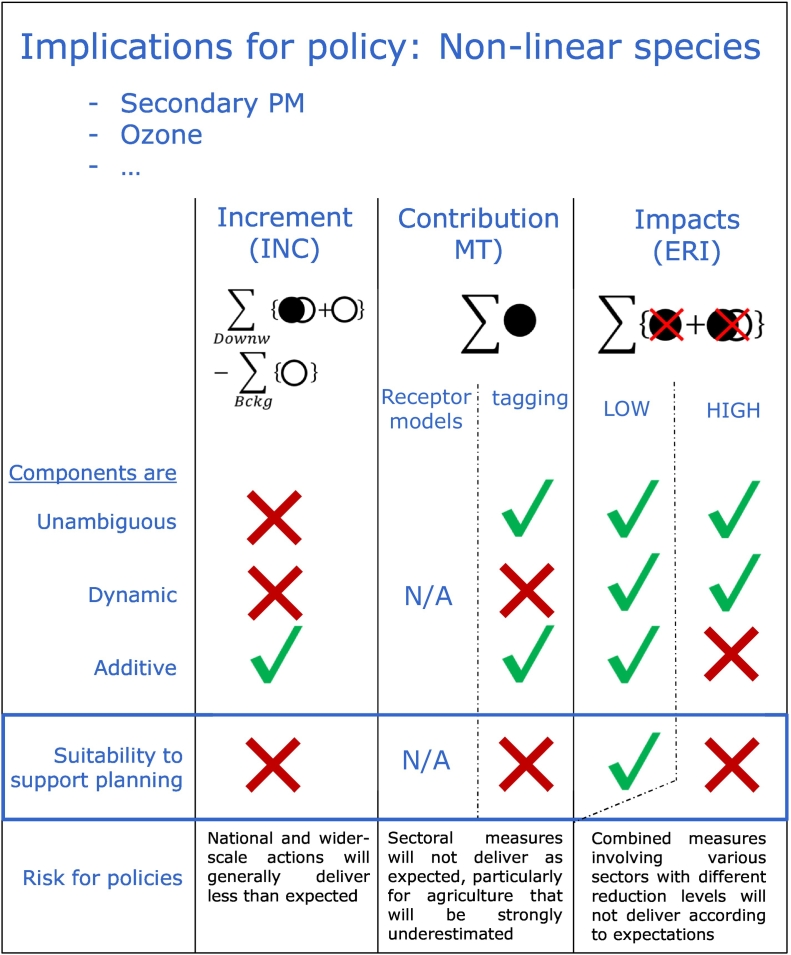
Criteria overview for source apportionment methods applied to non-linear species. The lowest two rows provide information on their suitability to support air quality planning and on potential risks for policy. Note: The tilted dashed line indicates that the risks for policy only apply to ERI-HIGH for this column.

For non-linear species, all approaches have limitations and shortcomings, which do not allow them to meet all criteria fully. However, some methods are intrinsically not suited for supporting air quality planning (design of mitigation strategies) because of their underlying assumptions. Fig. 11 summarizes the degree of fulfilment of the three criteria stated earlier for the different source-apportionment approaches.

Source allocation (ERI-LOW) produces *impacts* that are unambiguous, additive and dynamic. Although calculated for a specific fraction of the emission, the *impacts* remain valid over a certain range, ensuring robustness. While this is only valid for moderate emission reductions without drastic changes in the prevailing chemical regime, it is however often a realistic approach for policy makers as it is usually not possible to switch off entirely an activity in practice. For these reasons, source allocation is suited for supporting planning, but care must be taken to fix its range of validity. For higher fractions, ERI-HIGH is not suited for supporting mitigation strategies because of the non-additivity of the *impacts* as well as because of their lack of robustness.

Because *contributions* (MT-SM) lack dynamicity (no link with emission reduction *impacts*), the approach is not suited for supporting air quality planning when non-linear species are involved. Although in agreement with several other studies (Burr and Zhang, 2011a; Qiao et al., 2018; Mertens et al., 2018; Clappier et al., 2017; Grewe et al., 2010, Grewe et al., 2012), this is an important outcome of this work as tagging/labelling approaches are increasingly used in current applications to provide input to the preparation of air quality plans. This is the case, both for PM (Qiao et al., 2018; Guo et al., 2017; Itahashi et al., 2017; Timmermans et al., 2017; Wang et al., 2015; Hendriks et al., 2013) and for ozone (e.g. Borrego et al., 2016; Li et al., 2016; Wu et al., 2011). It is also the case for the TOPAS on-line platform https://topas.tno.nl/ and the LOTOS_EUROS source apportionment applications under CAMS/COPERNICUS (policy.atmosphere.copernicus.eu/CitySourceAllocation.html; Manders et al., 2017). All these applications use *contributions*, despite their recognized limitation for air quality planning applications.

One of the main reasons explaining the increased use of tagging/labelling *contributions* is probably its limited computational burden as *contributions* can be calculated with one single AQM simulation whereas *impacts* (ERI) requires a series of simulations to perform the same task. This is however, a surprising choice as alternative approaches are available that are more suited for air quality planning purposes and, at the same time, keep the computational burden relatively low, e.g. advanced sensitivity scenario methods such as the decoupled direct method: DDM (Dunker, 1984) or as the recent DDM based Path Integral Method (Dunker et al., 2019).

Finally, *increments* are generally not suited for both linear and non-linear species because their two additional underlying assumptions are frequently not fulfilled, resulting in ambiguous and non-dynamic components.

**Fig. 12:**
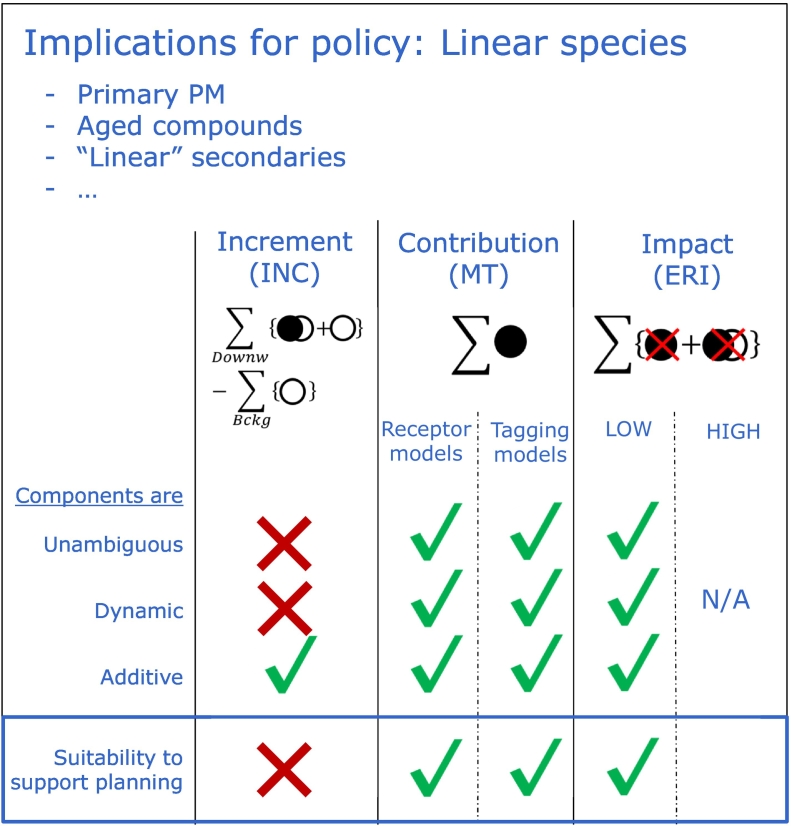
Overview of source apportionment methods and their degree of fulfilment of the criteria characterizing their components for linear species. The lowest row provides information on their suitability to support air quality planning.

For linear species, *contributions* (both MT-RM and MT-SM) are similar to *impacts* over the whole range of emission fractions apportioned. This is in agreement with all works dealing with this inter-comparison aspect (Kranenburg et al., 2013; Burr and Zhang, 2011b). An overview of the methods in case of linear species is presented in Fig. 12.

The implications discussed above result from the comparison among source apportionment results based on real-modelling data. It is important to note that because the three source apportionment methods are implemented in the same modelling set-up, the factor 2 or 3 differences highlighted in the results only arise from different conceptual assumptions. Differences are therefore not related to a possible lack of quality from the model or/and its associated input data.

## 6 Conclusions

Different approaches are available to policy makers to support them in the identification of sources responsible for air pollution levels and to inform on the efficiency of effective air quality plans. In this work, we reviewed three source apportionment methods: the incremental, the mass-transfer and emission reduction *impacts* (often referred to as brute force) approaches. The fact that these source apportionment methods generally serve the same purpose (support to air quality planning) but deliver different messages was analysed and discussed.

Both the theoretical and real-case modelling dataset presented in this work highlight the important differences among the three methods, in terms of result and therefore also in terms of their possible use for air quality planning purposes. These differences are a consequence of the assumptions that underpin the methodologies, with the main risk that these methods are sometimes used beyond their scope and range of applicability. In this work, we compared these three approaches with a focus on their specific use to support planning.

The comparative analysis led to the following conclusions:

1. *Increments*, either based on measured or modelled data, in general do not represent a suitable approach to support air quality planning. They might lead to large under- or overestimations of the actual responsibility of emission sources;

2. *Contributions* (mass-transfer approach) are appropriate to estimate spatial or sectoral *contributions* to support air quality planning only when the relation between emission and concentration changes remains linear throughout the entire reduction range (from 0 to 100%). This approach is not suited to support planning for non-linear species. One main mismatch is the quantification of the agriculture *contribution* that is largely underestimated with respect to the *impacts* as calculated with the ERI approach;

3. *Impacts* (ERI approach) are suited to support planning but it is important to assess carefully their application range (in terms of emission reduction). Given the well-known uncertainties attached to air quality modelling (incomplete emission inventories, gaps in the representation of atmospheric chemistry processes…), it is also important to assess the overall quality of the AQM for a given application. In this respect, both the *contributions* (mass transfer) and *increments* may be very useful for quality assurance purposes. For higher emission reductions, *impacts* are not suited because of their non-additivity and lack of robustness. Because of the level of non-linearities characterizing episodes, the issues raised here will be more acute for short-term than long-term air quality plans.

Due to non-linear processes, effective policies are not necessarily the ones tackling the most dominant emission source but those tackling the substance that is most scarce or binding in the pollution formation. This counter-intuitive result is difficult to communicate to policy makers. Neither the incremental approach nor the mass transfer approach will tell policy makers what measures are effective in reducing non-linear pollutants. Only simulation of various emission reduction scenarios will be able to support an effective policy strategy when non-linear processes are important. Of course, even that approach has limitations due the inevitable simplification in any model of chemical and meteorological processes, and weaknesses in emission and air quality data.

Although our conclusions are drawn from simple theoretical examples and from a unique dataset in one particular region (Po Valley), we believe them to be generally valid because differences in the results are driven by assumptions in the source apportionment methodologies that lead to systematic biases. For air quality planning, our findings show the need to consider carefully the choice of source-apportionment method. The biases and limitations of the different source-apportionment methods can explain why methodological choices may result in inefficient air quality control options.

## List of abbreviations

AQM: Air Quality Model
AQP: Air Quality Plan
ECA: European Court of Auditors
EEA: European Environment Agency
ERI: emission reduction impact
ERI-HIGH: emission reduction Impact for large emission reductions
ERI-LOW: emission reduction Impact for low emission reductions
EU: European Union
EU28: European Union (including the 28 Member States)
INC: incremental
M: mass
MT: mass transfer
MT-RM: mass transfer (receptor models)
MT-SM: mass transfer (source models)
PM: particulate matter
PPM: primary particulate matter
RC: regional contribution
RE: residual component/contribution
RI: regional increment
RIM: regional IMpact
UC: urban contribution
UI: urban increment
UIM: Urban IMpact
